# Decision-making for receiving paid home care for dementia in the time of COVID-19: a qualitative study

**DOI:** 10.1186/s12877-020-01719-0

**Published:** 2020-09-09

**Authors:** Clarissa Giebel, Kerry Hanna, Jacqueline Cannon, Ruth Eley, Hilary Tetlow, Anna Gaughan, Aravind Komuravelli, Justine Shenton, Carol Rogers, Sarah Butchard, Steve Callaghan, Stan Limbert, Manoj Rajagopal, Kym Ward, Lisa Shaw, Rosie Whittington, Mishca Hughes, Mark Gabbay

**Affiliations:** 1 Department of Primary Care and Mental Health, University of Liverpool, Liverpool, UK; 2 NIHR ARC NWC, Liverpool, UK; 3 Waterhouse Building B Block, University of Liverpool, Brownlow Street, Liverpool, L69 3GL UK; 4 Lewy Body Society, Wigan, UK; 5 Liverpool Dementia Action Alliance, Liverpool, UK; 6 SURF Liverpool, Liverpool, UK; 7 Together in Dementia Everyday, Liverpool, UK; 8 North West Boroughs NHS Trust, Warrington, UK; 9 Sefton Older People’s Forum, Sefton, UK; 10 National Museums Liverpool, Liverpool, UK; 11 Mersey Care NHS Trust, Liverpool, UK; 12 EQE Health, Liverpool, UK; 13 Lancashire and South Cumbria NHS Trust, Preston, UK; 14 The Brain Charity, Liverpool, UK; 15 Department of, Liverpool, UK; 16 Me2U Day Care Centre, Liverpool, UK

**Keywords:** Formal care, Dementia, COVID-19, Corona virus, Carers

## Abstract

**Background:**

The lockdown imposed in the UK on the 23rd of March and associated public health measures of social distancing are likely to have had a great impact on care provision. The aim of this study was to explore the decision-making processes of continued paid home care support for dementia in the time of COVID-19.

**Methods:**

Unpaid carers caring for a person living with dementia (PLWD) who were accessing paid home care before COVID-19 and residing in the UK were eligible to take part. Participants were interviewed over the phone and asked about their experiences of using paid home care services before and since COVID-19, and their decision-making processes of accessing paid home care since the outbreak and public health restrictions.

**Results:**

Fifteen unpaid carers, who were also accessing paid care support for the PLWD before COVID-19, were included in the analysis. Thematic analysis identified three overarching themes: (1) Risk; (2) Making difficult choices and risk management; and (3) Implications for unpaid carers. Many unpaid carers decided to discontinue paid carers entering the home due to the risk of infection, resulting in unpaid carers having to pick up the care hours to support the person living with dementia.

**Conclusions:**

This is the first study to report on the impact of COVID-19 on paid home care changes in dementia. Findings raise implications for providing better Personal Protective Equipment for paid carers, and to support unpaid carers better in their roles, with the pandemic likely to stay in place for the foreseeable future.

## Background

Dementia affects approximately 50 million people worldwide [1], and is characterised by difficulties with cognition, behaviour, motor performance, and everyday functioning, amongst others, all of which can vary by the dementia subtype [2, 3]. Depending on the symptom severity, unpaid carers (family members or friends) can also be affected by the dementia, by providing increased levels of unpaid care [4]. This in turn can cause higher levels of carer burden, therefore the needs of unpaid carers are of equal importance and should be supported in the dementia-caring process.

Generally, people living with dementia (PLWD) want to remain independent and living in their own home for as long as possible, without wanting to rely on others [5]. To enable living in the community for as long as possible, paid carers become a vital aspect of care for many PLWD as the condition advances. Home care can include both support with instrumental activities of daily living, such as helping with food preparation, and more basic activities of daily living, such as personal care with bathing and toileting or getting dressed. Needing support with personal care is usually more pronounced as the dementia progresses [6], and home care services are often employed when the PLWD becomes more dependent with daily activities [7] and/or the unpaid carer, a family member or friend, is unable to provide sufficient support [8]. However, across Europe, receiving formal care is often perceived as a threat to PLWD’s independence and thus only accessed if required [9].

While receiving home care is beneficial to both the PLWD and the unpaid carer, if available, there are many issues surrounding paid home care. Often, carers only visit for a short period of time to ensure medication adherence or getting a person in and out of bed for example, with little time for interaction with the PLWD [10]. Moreover, it is rarely the same paid carer who visits the home, but various different carers coming in each day, which can be difficult for the care recipient to build a rapport with the carers [11].

Since the first recorded case of COVID-19 in Wuhan, China, on the 31st of December 2019, COVID-19 has spread all across the globe and has had severe impacts on everyone’s life. To reduce the spread of the virus, severe public health restrictions have been put in place in most countries, which in itself can have a negative impact on people’s lives. In particular, COVID-19 related public health restrictions of lock down and social distancing can have a direct impact on the care they receive. Considering that the pandemic is going to lost for the foreseeable future, and face-to-face care needing to be adapted to continue being provided in a safe way, it is important how unpaid carers make decisions surrounding paid home care workers entering the home. Even post-COVID-19, it will be useful to understand how carers make risk management decisions for paid home care, as other pandemics or endemics might happen in the future.

The aim of this study was to explore the decision-making processes of unpaid carers in receiving paid home care for someone living with dementia in the time of COVID-19. To date, no research has explored the impact of COVID-19 on paid home care changes and adaptations, and how unpaid carers are making decisions about home care services. While the needs of PLWD are not subsiding and thus carers still need to provide care, this study will shed light on how unpaid carers judge the risk of strangers providing care versus providing care themselves, where possible. This study will provide vital and highly novel and topical evidence on how paid home care services are delivered and adapted in this pandemic, with findings having implications for continued paid home care provision.

## Methods

### Participants and recruitment

Unpaid carers (family members or friends) aged 18 or above were eligible to participate. For this analysis, we only included interviews from unpaid current carers who have had experiences of accessing, or trying to access, paid home care (*n* = 15).

Participants were recruited nationally via social care and social support services using convenience sampling. These support services included for example both regional and national carer and dementia organisations, who have a carers and PLWD receiving their newsletters, attend groups, and/or are linked in with other activities that create a network between carers and/or PLWD. To support recruitment, services provided information about the study in newsletters, and directly contacted eligible participants over the telephone to discuss the study. Contact details of eligible participants were forwarded to a researcher, who then contacted the participants.

We received ethical approval from the University of Liverpool Ethics [ID 7626].

### Data collection

Semi-structured interviews were conducted by telephone. The interview topic guide (Supplementary file) was co-developed with carers, PLWD, clinicians and academics. Data were collected between April and early May. Within this time period, a full nationwide lockdown was in place across the UK, with people only allowed to go outside once a day for essentials (food, medication) or for exercise. Vulnerable and older adults (to which younger people with dementia did not belong) were shielding, therefore recommended to not go outside at all and have food and other essentials delivered. No person was allowed to see anyone outside their households.

Carers were asked about their experiences of accessing social support services, including paid home-care services, before and since the COVID-19 outbreak. Participants were also asked about their demographic backgrounds, including age, gender, relationship to the person with dementia, postcode, years of education, type of dementia of the person they care for, as well as weekly hours of informal care before and since the virus outbreak. The interview guide is attached as Supplementary file.

### Data analysis

The overall recruited sample was 50 PLWD (*n* = 8) and unpaid carers caring for PLWD who lived with the carer (*n* = 23), who resided in a care home (*n* = 5), or who were living independent of the carer (*n* = 13). This sub-analysis on 15 interviews has been conducted from the interviews with unpaid carers only, who were solely responsible for the care of the PLWD (i.e. the PLWD was not residing in a care home during the time of the pandemic) and who were accessing paid care at the time of COVID-19, to specifically explore their experiences of accessing paid care. Interviews were conducted whilst participants continued to be recruited, allowing for recruitment to stop when the point of saturation was met, which occurred at 50.

Audio recordings were transcribed into verbatim scripts by a paid typist with extensive transcribing experience. Each transcript was anonymised and re-read for accuracy, allowing the authors to fully immerse themselves in the narratives before coding.

Transcripts were coded by all members of the research team (CG, MG, SB, LS, JC, SC, KH, KW). CG oversaw and allocated transcripts to each analyst. Line-by-line, manual coding of each transcript was employed individually by two researchers/trainees, and identified codes were discussed jointly to generate themes. Data were analysed using both inductive and deductive thematic analysis [12] by the eight research team members and trainee clinical psychologists. Of the 50 recruited participants, up to 35 transcripts were coded using inductive thematic analysis, which were discussed between transcribers. This generated the identified themes, with subsequent transcripts being coded using deductive thematic analysis to complement those themes.

## Results

Fifteen unpaid carers discussed their experiences of accessing paid home-care providers before and since the pandemic. All except one participant were female (93.3%), with most carers being spouses (53.3%). The majority (73.3%) of carers lived with the PLWD. The dementia subtypes of the PLWD, who were in receipt of paid care included, Alzheimer’s disease dementia (46.3%), vascular dementia (13.3%), and young-onset dementia without a specific dementia subtype (6.7%). Carers lived in a mix of disadvantaged and more affluent neighbourhoods, based on their IMD Quintile, with PLWD tending to live in more disadvantaged neighbourhoods. Participant demographics are summarised in Table 1.

**Table 1:** Demographic characteristics

	Carers in receipt of paid care (***n*** = 15)
Gender	
Female	14 (93.3%)
Male	1 (6.7%)
Ethnicity	
White	14 (93.3%)
BAME	1 (6.7%)
Relationship with PLWD	
Spouse	8 (53.3%)
Adult child	7 (46.7%)
Living with PLWD	
Yes	11 (73.3%)
No	4 (26.7%)
Dementia subtype	
Alzheimer’s disease	7 (46.3%)
Mixed dementia	2 (13.3%)
Vascular dementia	2 (13.3%)
YOD	1 (6.7%)
Other	3 (20.1%)
IMD Quintile	
1 (least disadvantaged)	3 (20.0%)
2	5 (33.3%)
3	2 (13.3%)
4	1 (6.7%)
5 (most disadvantaged)	2 (13.3%)
**Mean (SD) [Range]**	
Age	59.6 (+/−7.2) [45–74]
Years of education	15.0 (+/−3.2) [11–21]
Years since dementia diagnosis	6.5 (+/−4.7) [0.1–15]
Weekly hours of informal care before COVID-19	123.7 (+/− 72.7) [10–168]
Weekly hours of informal care before since COVID-19	124.5 (+/− 71.7) [0–168]

*BAME* Black and minority ethnic; *IMD* Index of Multiple Deprivation; *PLWD* Person living with dementia

Thematic analysis identified three overarching themes of (1) Risk; (2) Making difficult choices and risk management; and (3) Implications for unpaid carers. Further identified sub-themes are noted in Table 2.

**Table 2:** Coding tree showing results of thematic analysis

Theme	Subthemes
1. Risk	• Risk of virus transmission • Feeling unprotected and lack of Personal Protective Equipment • Feeling unprepared in providing additional care
2. Making difficult decisions and risk management	• Cancelling paid care • Keeping paid care • Avoiding hospitals/health providers
3. Implications to the unpaid carer	• Practical implications of food access • Personal implications and loss

### Theme 1: risk

#### Virus transmission and lack of personal protective equipment

Many unpaid carers reported feeling fearful of paid carers entering the homes of the PLWD and transmitting the corona virus. Furthermore, unpaid carers noticed changes to paid care support in terms of staff shortages. Instances occurred where paid care remained for some PLWD, although, often at a reduced level in order to adhere to social distancing measures, or where staff shortages resulted in limited time available with the PLWD. Fears of virus transmission increased due to the numerous, unfamiliar paid carers entering the home of a vulnerable PLWD.

*“I would imagine a lot of carers would [have] like been self-isolating themselves or for whatever reason that erm you know they would become short staffed and I didn’t like the idea of them, although bless them, going round caring for different people different, them going in someone else’s home and then going into my Mum I didn’t know who else they’d been in contact with …*”.

**Female carer (daughter), Interview 15.**

The frequent lack of personal protective equipment (PPE) further exacerbated the unpaid carers’ worries around transmitting the virus to the PLWD. In response to the question of whether the sole, unpaid carer of a PLWD, has been accessing any PPE, they replied:

*“no, I don’t and I haven’t got anything and to be honest I don’t think I’d be able to get hold of anything.”*

**Female carer (daughter), Interview 15.**

#### Feeling unprepared and providing additional care

Reports of the unpaid carers feeling unprepared in caring for the PLWD under these new, stringent circumstances emerged from the interviews. Unpaid carers described feelings of stress concerning the risk and fear of taking on extra caring responsibilities that they did not feel qualified to be doing. These accounts often stemmed from circumstances where the paid carers were new/unfamiliar with the PLWD, or where the level of available paid care support was limited, due to the above mentioned staff shortages.

“*… carers that needed us to sort of coach them really in how to care for Mum which we didn’t really know how to do … that meant that I needed to work [as] a carer that was essential because I didn’t know how to use all the equipment and I didn’t feel safe and also didn’t know how to protect myself from injury and well and being of risk to Mum.”*

**Female carer (daughter), Interview 21.**

### Theme 2: making difficult choices and risk management

#### Cancelling care

Many carers were faced with making difficult decisions in terms of cancelling their paid care support during the time of COVID-19. A central reason behind cancelling paid care stemmed from fears of transmitting corona virus and protecting the safety of the PLWD.

*“I ended up sitting down having a conversation with my Husband and my Dad to say, you know, we can’t have this many people coming in and out of the house especially people we don’t know. Erm so it was us as a family actually that contacted the care agency and said that not only would we erm deliver Mum’s care at teatime but I would erm take over Mum’s care at night time as well”.*

**Female carer (daughter), Interview 21.**

*“what used to be a help to keep him occupied while I was at work is now effectively my only respite time in the week. I get 3 hours on a Monday and 2 and a half hours on a Friday morning both of those have had, 1 of them I had stopped anyway because of the number of homes she’s visiting and I didn’t want to risk bringing anything back to [PLWD]”.*

**Female carer (spouse), Interview 31.**

#### Keeping care

Where paid home care remained constant for some PLWD during the pandemic, unpaid carers seemingly understood why others would want to cancel carers entering the home, acknowledging the accompanying risks in transmitting the virus. However, for these unpaid carers cancelling paid care was not an option due to the unimaginable burden it would leave them with. In these cases, the unpaid carers made the difficult decision to continue receiving paid care during the pandemic.

*“I couldn’t cope on my own and I’m just grateful to them [paid carers], that they still come … I don’t know what I would do if they didn’t because she’s not a big woman at all but she’s a dead weight and she, you know to, just to manage the personal care, one person couldn’t do it on their own. So no I obviously have thought about is it sensible to have them coming in and there’s an element of risk but I think it’s a risk I have to take.”*

***Female carer (daughter), Interview 27.***

Unpaid carers described real fears of re-obtaining future paid care (post-COVID) if they did make the difficult decision to cancel this support. Where the quality of paid care had dropped during the pandemic, this fear prevented unpaid carers of stepping in and taking on additional caring duties.

*“I am fearful and I know this has happened to a family err friend of ours who’s already been told by their social worker that because they’ve managed without the [paid] care then they’re not likely to get it back after the coronavirus and I’m quite fearful that will be the case for us because I don’t, I’m not able to do this fulltime”.*

***Female carer (daughter), Interview 21.***

*“I mean I know she’s got the carers going in and obviously carers will I mean I don’t know how the system works to be fair. But the Council, no doubt, will be in touch with the care company that I use, so, but that’s, to me, that’s just the money side of things. I don’t know, every April I get in touch with the Council and I say can I have a copy of my Mum’s care plan because obviously getting the direct payment that I get I have to work out what the top fee is etc and I’ve had to really ring on numerous occasions just to get a copy of the care plan just so that I can work the money side of things out correctly …*”.

***Female carer (daughter), Interview 15.***

#### Avoiding hospitals and health providers

Many respondents expressed a fear of the PLWD being admitted to a hospital due to fears of contracting the virus, whilst further expressing gratitude that they were still living at home and not being cared for in a care home following negative media reports. Unpaid carers attempted to manage health concerns at home, to avoid coming into contact with hospitals and health providers.

*“I’ve got a prescription … for antibiotics if erm if I was to need it now rather than erm, I don’t know, if that would help frankly but anyway (laughs) erm its better than you know with, the last thing I want is for her to go into hospital. And I’m extremely grateful that she’s not in a care home.”*

***Female carer (daughter), Interview 27.***

### Theme 3: implications for unpaid carers

#### Practical implications of food access

A common theme that emerged from the interviews was the inability to provide for the PLWD, as carers were unable to successfully shop online, with many experiencing technical difficulties and poor access to grocery webpages. In this pandemic, people aged 65+ or with health conditions that make them more prone to suffer from the virus are placed on a ‘vulnerable list’ by the government in the UK. This allowed people to get priority shopping slots compared to the general population, and get food delivered to their home, as they were not supposed to leave the house even for grocery shopping. A common complaint underpinning this issue was that dementia failed to be classified as a “vulnerable” group, thus limiting access to priority shopping slots.

“*… of course a lot of the things that I’d normally do, I normally do online shopping, I place an order, it arrives, I put it away, job done, it’s not a stressor. At the moment I can’t get an online shopping slot. Trying to get through to the helpline to get us put on the vulnerable list has proved an impossibility, I’ve spent hours and hours and hours on the phone which gives you a layer of angst that on top of everything else you don’t need you know”.*

**Female carer (spouse), Interview 31.**

*“I couldn’t get a shop in online and I think that’s been the biggest thing with me.”*

**Female carer (spouse), Interview 29.**

Where shopping slots were available for some respondents, they indicated that this was due to pre-existing health conditions that classed them as “vulnerable”, such as pulmonary or cardiological impairments, but not the dementia. This resulted in frustration and a fear of entering shops due the risk of transmitting the virus.

*“I’m very frustrated with the Government that Alzheimer’s or dementia isn’t classed as a vulnerable person because I can’t get a slot for the shopping and that’s, we haven’t had a letter or anything and without this letter with a code number on you can’t get priority … I’ve been every day I go on to see if I can get a slot from any of the supermarkets … I daren’t go shopping I just daren’t go shopping … they’re the most dangerous places to go from the sounds of things”.*

**Female carer (spouse), Interview 13.**

Unpaid carers that could not shop online, and therefore had to risk entering stores, faced an added dilemma, as they were unable to leave the PLWD home alone during that time. This situation caused further complications and health concerns for the PLWD, due to the dementia-related cognitive impairment and inability to comprehend and abide by social distancing measures, increasing stress and carer burden.

*“he doesn’t understand the thing about you know like keeping the distance when you’re out and that … ‘cause they’re saying you know it’s one person per shopping trolley I just show I’ve got a carers card from like [residential area] Council and I say I can’t leave him on his own …*”.

**Female carer (spouse), Interview 41.**

#### Personal implications and loss

Many unpaid carers made drastic changes to their own lives to support PLWD during COVID-19, including cancelling paid care, moving in with the PLWD and working from home (their paid employment) whilst caring full time. This led to increased workload and strain on the unpaid carer. This strain is heightened with fears of uncertainty and the long-term commitment to caring.

*“at first I thought, “oh this is really good” obviously because I was working from home it was much easier and Mum was having good days as well you know easier to get her out of bed, willing to get out of bed. But these last few days she’s been what I call her bad dementia days where she’s a little bit more difficult and obviously I’m getting more tired but it’s just something I want to do for my Mum at the same time but yes you think obviously as time is going on it’s becoming harder.”*

***Female carer (daughter), Interview 15.***

In addition to the marked increase in caring responsibilities due to the loss of paid home care, unpaid carers noted a loss of family and peer support due to the social distancing measures. This loss further added to their feelings of stress and fatigue in caring solely for the PLWD at a higher level than before COVID-19.

*“so he [son] won’t come anywhere near [PLWD] in case he’s a shedder. So I’m panicking if this doesn’t go right … who’s going to look after John now because they [sons] won’t come anywhere near because they’re frightened that they will pass the virus onto us”.*

***Female carer (spouse), Interview 29.***

Even where unpaid carers retained some paid support during the time of COVID-19, this was often minimal, and at times ineffective. A phone call from a social worker was appreciated but ultimately, could not offer any practical support to a family who are struggling with the burden of caring.

*“we have heard from our social worker who is fantastic and just to sort of have a phone conversation about [how] we were coping … she said we’re going to be there support you is there anything we can do for now? But there’s nothing they can do”.*

***Female carer (daughter), Interview 21.***

## Discussion

This is the first study to show the impact of COVID-19 public health restrictions on receiving paid home care in dementia. Unpaid carers had to make difficult choices on whether to continue paid home care, and where it was discontinued, experienced a strong impact on their lives as carers.

Paid home care is often characterised by numerous different carers entering the home, with families however valuing consistency in care workers [13]. In light of COVID-19, receiving a large number of different carers each week can increase the risk of virus transmission into their home directly by contamination of surfaces or by providing basic care in close proximity to the PLWD. This caused a great deal of worry to unpaid carers, as they had no knowledge of whether individual paid home care workers adhered to public health restrictions or met up with members of different households after work. Specifically, where care workers fail to bring and use suitable PPE, many carers experience distress and feel helpless. With a severe shortage of PPE in the UK already in the healthcare sector, including hospitals, the social care sector is likely to face even more shortages, despite the great focus on some of the most vulnerable of our society. PPE usage reported by carers was not always consistent, which is in line with a recent review on PPE recommendations and usage [14]. Therefore, to enable unpaid carers to continue receiving paid home care, the care sector needs to be better, and consistently, equipped with PPE. With a considerable emphasis in the news on the increased risk for chronically ill and older people, this further caused heightened levels of fear of likely fatal outcomes from COVID-19 infections.

Carer burden is already a large problem in dementia care, with family members and friends providing an estimated £14 billion of care each year in the UK alone [15]. Levels of burden increase with increased symptomatology as the dementia progresses [16]. Considering that paid home care is supposed to take some of the caring duties away from unpaid carers, suddenly having to pick up additional caring duties due to COVID-19 restrictions and risk management decisions can place an additional layer of burden onto the carer. Carers can in certain ways be supported better by accessing other forms of social support services, such as peer support groups or respite care [17]. However, due to the pandemic, these services have been severely affected also, not offering the once provided benefits for many people any more and resulting in reduced levels of well-being for carers. Therefore, other ways of supporting carers need to be found, to ensure that carers do not have to suffer from increased levels of burden and poorer mental well-being.

While some unpaid carers discontinued paid home care, others accepted the potential risks posed by non-household members (care staff) entering the home. Some carers needed the support from professional carers in looking after the person with dementia, and would have been unable to cope otherwise. Carers thus made risk management decisions, which follow recently highlighted risk management strategies by Gasmi and colleagues [18], highlighting the need for individual assessment of risk factors for example based on medical conditions, gender, age, lifestyle, and environmental risks. Managing the risk of contracting COVID-19 thus needs to be based on individual circumstances and cannot be a one-size fits all approach. Whilst these unpaid carers might have been more accepting of carers entering the home, care staff still needs to be supported better in providing home-based care, allowing more families feeling comfortable of receiving much needed support from care staff.

This study also had some limitations. We only interviewed unpaid carers and their experiences of accessing paid home care for their relative with dementia in the time of the pandemic. However, we did not interview paid home care staff, and the effects of providing care in clients’ homes in light of COVID-19, potential lack of PPE, and the risks of potentially becoming infected have to date not been explored. In England and Wales, social care staff are some of the employment groups experiencing some of the highest rates of COVID-19 related mortality [19]. Moreover, a recent rapid review showed that health care staff working with patients in novel virus outbreaks are at increased risk of suffering psychological distress when being younger, as well as having a family member infected by the virus, with adequate PPE, rest, and support reducing morbidity [20]. Future research needs to explore the effects on social care staff and care workers specifically and their experiences and impacts on psychological well-being of providing care in the time of COVID-19, as well as care home staff where very limited evidence exists to date [21]. Moreover, whilst this study is representative in terms of gender distribution, with women representing the largest proportion of unpaid dementia carers globally [22], future research should explore specifically the perceptions of male unpaid carers towards risk management decisions in receiving paid home care during this pandemic.

## Conclusions

This is the first study providing important insights into the decision-making processes and experiences of receiving paid home care for dementia in the time of the pandemic. Paid home care is significantly affected by family members’ decisions on whether care should be continued or not. Considering the increased burden on unpaid carers where decisions are made to discontinue care, improved guidance and logistical support needs to be in place for care staff, in terms of access to and proper usage of adequate PPE, and potentially reducing the numbers of care staff entering the home, to further minimise the risk of potential virus transmission. This could be partly facilitated by technology, for example providing updates on care plans and which staff members are currently off sick with the virus. This is a significant concern considering the close proximity in which care is usually provided, by showering, dressing, and supporting the PLWD to use the toilet. With PLWD being less likely to entering a care home in the current climate of the pandemic, due to further increased risk of virus transmission, enabling easily accessible and safe paid home care is becoming even more important to support PLWD living at home.

## Supplementary information

**Additional file 1 Supplementary file 1**. Interview topic guide

## Data Availability

The datasets generated and analysed during the current study are not publicly available due ethical approval only allowing storage of transcripts on the University of Liverpool hard drive, but are available from the corresponding author on reasonable request.

## Abbreviations

PLWD: People living with dementia
PPE: Personal protective equipment
