# Supplementary material for: Decision-making for receiving paid home care for dementia in the time of COVID-19: a qualitative study
Source: BMC Geriatr. 2020 Sep 9;20:333. doi: 10.1186/s12877-020-01719-0 (PMC7478902; doi:10.1186/s12877-020-01719-0)
Supplement: Supplementary file 1 — Additional file 1 Supplementary file 1. Interview topic guide [file 12877_2020_1719_MOESM1_ESM.docx]

**Supplementary file 1. Interview topic guide**

Q1. We are interested in your experiences surrounding dementia care in general. What types of services have you and the person you care for been accessing since the dementia diagnosis before Covid-19?

Q2. Are you aware of any services available: which, and how did you find out about them?

Q3. Do you have to pay to access these services, or how are they funded?

Q4. What are the effects of using these services? How do they make you and the person you care for feel?

Q5. Since the corona virus outbreak, day care centres, support groups, and other social care services have been temporarily shut down. How did that make you feel when you first found out? How much does this affect you in your daily life as a carer?

Q6. How do you feel about your relative (and you) having to self-isolate (if late-onset dementia or other chronic conditions and or >70)? In what ways does it affect you, and do you have to adapt the care you provide? What about more generally in terms of isolation and being confined to the home? What might help?

Q7. Do you access any digital support to help you care for your relative?
